# Yield growth patterns of food commodities: Insights and challenges

**DOI:** 10.1371/journal.pone.0313088

**Published:** 2024-11-27

**Authors:** John Baffes, Xiaoli Etienne

**Affiliations:** 1 World Bank, Washington, D.C., United States of America; 2 Department of Agricultural Economics and Rural Sociology, University of Idaho, Moscow, ID, United States of America; University of Georgia, UNITED STATES OF AMERICA

## Abstract

Understanding global food production and productivity patterns is crucial for policy and investment decisions aimed at addressing poverty, food insecurity, and climate change. This paper develops comprehensive calorific-based production and yield indices for 144 crops, covering 98% of global agricultural land and food output. These indices provide standardized measures across various crops and varieties, facilitating comparison of agricultural productivity and consolidating country and regional contributions to global food production. Utilizing a Box-Cox transformation, we find that a linear model best approximates yield growth. Our findings reveal that, at an aggregate level, there has been no discernable slowdown in global yield growth over the past six decades. This translates into an average annual yield increase equivalent to nearly 33 kilograms of wheat per hectare. These results suggest that any observed deceleration in specific commodities, regions, or countries, has been offset by gains in others. While these findings are reassuring from a global food supply perspective, caution is warranted about the sustainability of production and the affordability of food. These concerns are particularly relevant as global food demand increases due to population and income growth, and as the pressures from climate change intensify. The study underscores the importance of adopting strategic and sustainable agricultural practices to ensure continued food security in the face of evolving global challenges.

## 1. Introduction

As the global population approaches 10 billion by the mid-century, agricultural productivity will become increasingly critical in feeding the world [1]. Over the past six decades, productivity improvements have accounted for much of the growth in food production. However, the rate of yield growth for food commodities, a key measure of productivity, has been perceived to have stagnated in recent decades [2–5]. This assessment has led to concerns regarding food availability, especially in low and middle-income countries where population growth rates are the highest, including Sub-Saharan Africa [6]. The slowdown has also been cited as a cause of commodity price spikes and price volatility [7].

Traditionally, yield growth patterns have been analyzed using single-commodity, weight-based data (e.g., kilograms or metric tons per unit of land) at country, regional, and global levels. While the bulk of the empirical studies finds widespread yield growth deceleration or stagnation [1, 4, 8], closer examination reveals considerable heterogeneity across crops and regions. Notably, high-income countries that experienced large yield gains in response to the Green Revolution earlier in the 20^th^ century appeared to have experienced stagnation or even deceleration. Certain low-income countries, especially Sub-Saharan Africa, also faced similar problems due to limited access to high-yielding varieties and production inputs. However, contrary to the stagnation and deceleration narrative, yield growth acceleration has been observed for various commodities and regions [9].

This heterogeneity in yield growth patterns across crops and regions raises two crucial research questions: Firstly, has aggregate global crop yield experienced a slowdown or stagnation? Secondly, how similar or dissimilar are yield growth patterns across commodities, countries, or regions? Although single-commodity models are useful for identifying supply-driven issues such as the effects of weather patterns, climate change, or technological improvements for a specific crop, they do not provide a comprehensive assessment of aggregate yield dynamics across diverse crops and regions. Indeed, analyzing sustainability issues and addressing food security concerns requires a modeling framework that allows us to evaluate the aggregate growth patterns not only across the entire food crop spectrum, but also accounts for changing crop patterns that may be driven by input costs, domestic and trade policies, as well as demand-side considerations, including changes in tastes and preferences.

Against this background, the present paper addresses the two research questions by introducing an innovative calorific-based approach to tackle the limitations of the existing literature. Instead of traditional weight-based measures such as metric tons or kilograms, we convert annual crop production into calorific content and subsequently aggregate the production and yield of all food commodities into single metrics. This aggregation accounts for heterogenous yield patterns due to changes in production composition, the transition from low-yield to high-yield crops and varieties as well as shifts in country and regional significance. Additionally, this approach provides an easy-to-implement, yet standardized and universal framework for analyzing and comparing yield growth at any level, from individual crops to commodity groups to global aggregates.

Using these calorific-based indices, we apply statistical methods to select the most appropriate model for charting the yield paths of commodities at global, regional, and crop-group levels. Production and calorific content data for 144 major crops covering the 1961–2021 period from the Food and Agricultural Organization (FAO) are included in the analysis. These crops combined account for approximately 98 percent of the world’s agricultural land area. The evidence suggests that the aggregate global yield index has not been subjected to growth deceleration over the past six decades. Thus, slow growth in certain commodities, regions, or countries, documented in the literature has been offset by accelerated growth in others.

The remainder of the paper proceeds as follows. The next section summarizes the literature on yield growth. Section 3 discusses the aggregate yield index, the modeling framework for evaluating yield growth, and the data. Section 4 presents the results. The last section concludes and discusses avenues for further research.

## 2. A brief review of literature

The literature assessing yield growth performance can be broadly delineated into three principal strands. One strand delves into yield growth from experimental data, serving as a tool for plant scientists to discern performance nuances and facilitate the selection of crop varieties [10–12]. The second strand focuses on evaluating the statistical distribution of yields, with the explicit objective of assisting farmers to make informed selection of crop varieties, while also aiding the insurance industry in determining premia for potential crop losses. Pioneering contributions to this strand include an early study by Day [13], subsequently expanded upon by Just and Weninger [14], Atwood, Shaik and Watts [15], Norwood, Roberts and Lusk [16], and Sherrick et al. [17]. The third strand, particularly pertinent in the present context and the subject of this section, examines yield growth through the lens of sustainability and food security considerations.

Under this strand of literature, determining whether yield growth has encountered deceleration or stagnation has been a topic of substantial debate with most studies focuseing on grain and oilseed crops. At the global level, Alston, Beddow and Pardey [2] conducted a comprehensive study documenting a discernible slowdown in the growth of grain yields. Their findings underscored the potentially far-reaching implications for food price trends should this deceleration persist. Cassman [18] contributed to this discourse by highlighting that the rate of yield increase for cereal crops potentially fell significantly short of the anticipated rise in food demand. Examining the trends in yields for the Big-4 commodities (maize, soybeans, wheat, and rice), Ray et al [5] concluded that, while yields continue to ascend in many regions, a noteworthy proportion—ranging from 24% to 39% of the Big-4 growing areas—exhibits a concerning pattern of either negligible improvement, stagnation, or outright collapse. Subsequent research by Ray et al [1] revealed that the growth rates of yields in these commodities fall considerably short of the levels required to meet the projected demand for food commodities by 2050.

At the regional level, Van Ittersum et al [6] asserted that the stagnation of yields in Sub-Saharan Africa presents a formidable food security risk, particularly as the population of the region is anticipated to reach 2.1 billion by 2050 (from 1.4 billion in 2020). Using grid-level data, Iizumi et al. [19] found increased yield instability for the Big-4 commodities across a broad region of the Southern Hemisphere, corroborating previous findings of yield stagnation and collapses in these regions. Lin and Huybers [20] examined wheat yield data across 47 regions, finding that approximately half of the production within their sample continued with a linear growth trend, while the remainder exhibited yield stagnation. Michel and Makowski [21] estimated that wheat yield growth exceeded 0.06 tons per hectare per year in 1961–2010 in various countries across Europe, Asia, Africa, and America, but it experienced stagnation in many other countries. Focusing on 24 African countries from 1960 to 2012, Saito et al. [22] found that 15 countries experienced accelerating yield growth, while the rest saw stagnation or decline.

Numerous studies examined country-level crop yield growth as well. In a focused analysis of China’s yield performance spanning 1980–2010, Li et al [8] observed yield stagnation in 50% of rice-producing areas, 54% of maize-producing areas, and nearly 16% of wheat-producing areas. In the context of analyzing food security in China, Wei et al. [23] determined that rice yields face pronounced stagnation in 53.9% of the regions examined, followed by 42.4% in maize and 41.9% in wheat. Finger [9] found that while the yield growth of maize, barley, and rye remained at a linear trend in Switzerland from 1961 to 2006, other commodities, including wheat, experienced yield growth deceleration. Examining yields in 29 Indian states for the 1967–2017 period, Madhukar, Kumar and Dashora [24] found that 76%, 47%, and 18% of the harvested areas did not show yield improvement in the recent decade for wheat, rice, and maize, respectively.

Notwithstanding the prevailing, somewhat pessimistic, outlook in the studies reviewed above, it is essential to note that not all research aligns with such a perspective. In an influential work, Alexandratos [25] contended that global agricultural production is poised to meet, or potentially exceed, the demands of food requirements. Alexandratos also emphasized that the critical concern lies in the persistent challenges of poverty in low-income countries. Similarly, an editorial in the journal *Nature* [26] offered a nuanced perspective on productivity growth and food security, noting that depending on research advancements in the sector, it is feasible to meet the 2050 global food demand at an acceptable cost. Ausubel, Wernick and Waggoner [27] echoed similar views, highlighting the substantial increase in crop yields over the past 50 years, alongside a marked decline in caloric requirements relative to GDP.

To summarize, the existing literature provides mixed evidence on yield growth trends, with findings varying based on the specific commodities, countries, regions, and time periods analyzed. While some studies indicate a decline or stagnation for certain crops and regions, others report an acceleration or consistency with a linear growth trend. This raises a critical question: from a global perspective, has aggregate crop yield experienced stagnation or deceleration? Most existing studies have predominately employed weight-based yield to analyze productivity growth for single commodities. However, this approach does not adequately capture the rate of productivity change across all combined commodities, which is crucial for analyzing food security and sustainability challenges. In this study, we propose constructing global yield growth indices based on the calorific content of crops to assess aggregate yield growth patterns at the global, regional, and commodity levels. These patterns are discussed in the following section.

## 3. Methods and data

Indices have been used in various contexts within commodity markets, such as the aggregation of various commodity prices into a single index and the construction of aggregate agricultural productivity indices. A critical issue in the aggregation process is the selection of appropriate weights. For instance, the weights used in the World Bank’s commodity price indices are based on the export values of emerging markets and developing economies [28]. For agricultural productivity studies, weights are typically based on values derived from FAO international crop prices, measured in Geary-Khamis dollars per ton, also known as “international dollars” [29]. Numerous studies have employed FAO-based aggregation, including Adamopoulos and Restuccia [30] who studied cross-country agricultural productivity based on micro-plot level data; Gollin, Lagakos and Waugh [31] who examined agricultural productivity differences across countries for maize, rice, and wheat; Mekonnen et al. [32] who investigated technical efficiency in developing countries; and Nin-Pratt, Yu and Fan [33] who compared agricultural productivity growth in China and India.

However, using weights based on values derived from commodity prices for specific years can introduce bias for several reasons. Firstly, while there are established international price sequences for key agricultural commodities such as the Big-4, most other commodities, which are less frequently traded, lack benchmark prices. Second, in many low-income countries, geographical isolation and high transportation costs render many agricultural commodities non-tradable [34, 35]. Third, trade distortions often cause domestic prices to diverge from international benchmarks [36]. Additionally, many commodity prices are subject to long-term trends, medium-term cyclicality, and short-term variability, implying that selecting different sub-periods can yield varying results [37]. Therefore, yield aggregation based on value terms, even when adjusted for inflation or purchasing power, can suffer from rapidly changing weights due to price volatility. To address these issues, we employ calorific-based weights in our construction of global and regional production and yield indices.

Calorific-based indices have been widely used on the consumption side in various contexts, such as calculating food requirements for balanced diets and estimating budgetary needs to ensure healthy nutrition [38, 39]. However, their application on the production side has been less frequent, though they have been utilized in a range of contexts. Dating back to 1942, Williamson and Williamson [40] used calorific content of food commodities to discern patterns in food consumption. Roberts and Schlenker [41] extended this approach by converting the production of the Big-4 commodities to identify supply and demand elasticities in the context of the 2007–08 price spike. In a similar vein, Bobenrieth, Wright and Zeng [42] calculated stocks-to-use ratios for major grains and an index of total calories from these grains, serving as indicators of vulnerability to food shortages and price spikes. D’Odorico et al. [43] contributed to this line of inquiry by calculating the water intensity of internationally traded food commodities, employing a calorific aggregation. Additionally, Cassidy et al. [44] leveraged the calorific content of food commodities to assess area requirements for human food consumption.

Our study extends this strand of literature to evaluate aggregate crop yield growth. The remainder of this section details the construction of an aggregate yield index and discusses the key assumptions underlying common yield growth estimation approaches. It also examines the modeling framework used to select the appropriate specification for growth rate estimation, addressing how to manage structural breaks and nonlinearities. Additionally, it provides a brief overview of the data used in the analysis.

### 3.1 The aggregate yield index

The aggregate calorie-based yield index, *y*_*t*_, for a particular country/region is computed as:

$$y_{t}=\sum_{i=1}^{N}w_{i}Q_{it}/\sum_{i=1}^{N}L_{it}$$ (1)

where *Q*_*it*_ denotes the total output of commodity *i* at year *t* in weight unit; *w*_*i*_ represents the calorific content of commodity *i* per weight unit; and *L*_*it*_ is land allocated to commodity *i* at year *t*. Thus, *y*_*t*_ represents the amount of calories produced per land area (in *hectare*). In addition to a given country, the index can be constructed for the entire world or any region or aggregate commodity group of interest.

Common calorific units used include *cal* (small calorie) and *Cal* (large calorie). A small calorie is defined as “…*the amount of heat required to raise the temperature of 1 g of water by 1°C with a temperature change from 14*.*5 to 15*.*5°C*. *The current US Dietary Reference Intakes define 1 cal as 4*.*186 J [joules]*” [45]. One large calorie is equivalent to 1,000 small calories, or 1 *Cal* = 1,000 *cal*. Often *Cal* is denoted as *kcal*, a notation used in food labeling. For notational convenience, the present paper uses *KCa*l and *MCal*, which equal 1,000 large calories and 1,000,000 large calories, respectively. In other words, we define 1 *KCal* = 1,000 *Cal* = 1,000 *kcal*, and 1 *MCal* = 1,000 *KCal* = 1,000,000 *Cal*.

The benefits of the calorific-based approach over traditional weight-based methods can be illustrated with an example of two commodities that followed different production and yield paths. Millet experienced a modest increase in global production from 25.7 MMT (million metric tons) in 1961 to 30.1 MMT in 2021. The area allocated to millet during this period declined by almost a third, leading to an annual yield growth of 6.3 *kg/ha*. During this same period, maize production increased sixfold while the area doubled, giving an annual yield growth of 65.6 *kg/ha*. To assess the combined yield growth performance for these two crops, production is converted from metric tons to calories (3,400 *kcal/kg* for millet and 3,560 *kcal/kg* for maize) and aggregated into a single metric by adding their global calorific production. The annual calorific-based yield growth for the two commodities combined is 219 *KCal/ha*. This calorific approach allows us to assess the aggregate yield growth of two crops with different growth paths in a way that the traditional weight-based approach cannot.

### 3.2 Assumptions under traditional methods of estimating yield growth patterns

The growth rate between periods 1 and 2, denoted by *ρ*, typically reported as percent change, is calculated as *ρ* = (*y*_1_−*y*_0_)/*y*_0_, where *y*_1_ and *y*_0_ denote yield in the current and previous periods. The growth rate equation can also be written as *y*_1_ = (1+*ρ*)*y*_0_. In a multiperiod case, if yield grows at rate *ρ*, on average, while in each period it is subjected to a stochastic shock *η*_*t*_, yield at time *t* can be rewritten as *y*_*t*_ = (1+*ρ*)*y*_*t*−1_ *η*_*t*_.

Taking a one-period lag results in $y_{t-1}=(1+\rho )y_{t-2}\eta_{t-1}$, which upon substitution and working backward to period 0 results in $y_{t}=(1+\rho {)}^{t}y_{0}\eta_{t}\eta_{t-1}\ldots \eta_{0}$. Taking natural logarithms of both sides and setting *β*_0_ = *log*(*y*_0_), *β*_1_ = *log*(1+*ρ*), and $\varepsilon_{t}=\sum_{i=0}^{i=t}\log(\eta_{i})$, gives: $\log(y_{t})=\beta_{0}+\beta_{1}t+\varepsilon_{t}$. Such logarithmic transformation is a frequently used regression where the parameters *β*_0_ and *β*_1_ are estimated with ordinary least squares and the growth rate is calculated as *ρ* = *exp*(*β*_1_)−1. The growth rate is often reported as the estimate of *β*_1_ rather than *ρ*, since for small growth rates *β*_1_ and *ρ* are approximately equal. Estimating growth rates by using logarithmic transformations rests on the assumption that *y*_*t*_ grows at approximately the same rate (in percent terms) throughout the sample period, in order to render the error term white noise (*η*_*t*_ is log-normally distributed with mean 1). This model essentially assumes that yield follows an exponential growth pattern.

While the proportionality assumption may be a reasonable approximation for short periods (e.g., a decade) and relatively small growth rates, it may be unrealistic when long periods are considered, such as a 60-year sample in the current context, due to the change in the base. An alternative and, perhaps, more realistic, assumption could be that *y*_*t*_ grows by a constant amount in each period, say *μ*, in which case the two-period yield growth would be *y*_1_ = *μ*+*y*_0_ or, in the general case, *y*_*t*_ = *μ*+*y*_*t*−1_. For the multi-period case, backward substitution gives *y*_*t*_ = *μt*+*y*_0_. Letting *β*_0_ = *y*_0_ and *β*_1_ = *μ*, and appending an additive error term gives *y*_*t*_ = *β*_0_+*β*_1_*t*+*ε*_*t*_. This linear growth specification implies that yield grows at an average rate of *β*_1_ units.

The fundamentally different nature of these specifications, i.e., exponential vs. linear growth models, has important implications for assessing whether growth has decelerated or accelerated. To illustrate, consider that maize yield grew at 2.6 and 1.7 percent per annum during 1961–71 and 2011–21, the first and the last decade of the sample. However, maize yields grew at 203 KCal and 324 KCal annually during the same periods. In other words, yield growth for maize has dropped by more than one-third (based on the logarithmic specification that assumes a constant percentage growth) but has increased by more than 50 percent (based on the linear specification that assumes a constant amount of growth).

### 3.3 Choosing the appropriate specification

The discussion above suggests that yield growth should be modeled under a more general framework. We begin wih the following specification:

$$y(\lambda )=\beta_{0}+\beta_{1}t+\varepsilon .$$ (2)

where *β*_0_ is constant, *β*_1_ indicates the rate of yield growth, *ε* is *iid (independent and identically distributed)* error term, and *λ* is a transformation parameter, such that *y*(*λ*) is either *log*(*y*) (for logarithmic specification) or *y* (for linear specification). In this paper we employ the Box-Cox model to determine which specification best represents the data generation process. This approach selects the transformation of the dependent variable such that the residuals approximate a normal distribution and exhibit reduced heteroskedasticity [46]. Sakia [47] provides a comprehensive review of the Box-Cox transformation technique. Notable advancements to the Box-Cox transformation include Yeo and Johnson [48] transformation, which accommodates both positive and negative observations, and the extension by Atkinson, Riani and Corbellini [49], which allows for transformations on both sides of the equation.

The Box-Cox model relies on estimating the transformation parameter *λ* such that:

$$y(\lambda )=\left\{ \begin{array}{cc} (y^{\lambda }-1)/\lambda & \lambda \neq 0\\ log(y) & \lambda =0 \end{array}.\right.$$ (3)

All notations are the same as defined previously. Eq (3) embeds linear (*λ* = 1) and logarithmic (*λ* = 0) transformation of the dependent variable. Under the assumption that there exists a *λ* that makes the error term in the model approximately normal, Box and Cox (1964) derived the likelihood function for a set of observations {*y*_1_,*y*_2_,…,*y*_*t*_} and suggested using the maximum likelihood estimation (MLE) to determine *λ*. Further details on the MLE and alternative estimation procedures are discussed in Spitzer [50].

Regardless of the value of *λ*, Eq (2) assumes that the underlying parameter estimate of the growth rate is constant over time. However, yield paths may exhibit non-linearities, with growth rates gradually accelerating, decelerating, or even taking sharp turns. We consider two ways to account for such non-linearities. First, following earlier studies [e.g., 9], a squared time trend is added to Eq (4):

$$y(\lambda )=\beta_{0}+\beta_{1}t+\beta_{2}t^{2}+\varepsilon_{t},$$ (4)

where *β*_1_ approximates the growth rate and *β*_2_ denotes the rate at which growth decelerates (when negative) or accelerates (when positive). Higher-order polynomials could be included to capture more complex non-linearities. In Eq (4), *ε*_*t*_ again is assumed to be *iid* and other notations are defined as previously.

A second way to account for non-linearities is to impose one or more structural breaks and estimate separate growth rates for each subperiod. In the case of one structural break at year *τ*, a piecewise linear regression model can be utilized to estimate the pre- and post-break growth rates, while ensuring continuity at the break date [51]:

$$y(\lambda )=\beta_{0}+\beta_{1}t+\beta_{2}(t-\overset{˜}{\tau })D+\varepsilon_{t},$$ (5)

where $\overset{˜}{\tau }$ ia the estimated break year, *D* is a dummy variable taking the value of one for the years after the break and zero otherwise, *ε*_*t*_ is *iid* error, and other notations are defined as previously.

Since we have no *a priori* knowledge of the existence of a structural break, we use the Quandt Likelihood Ratio (QLR) procedure to determine if (and when) the data-generating process has been subjected to breaks [52]. Specifically, a sample Wald test statistic is computed for every possible break date (*τ*) to determine the stability of parameters before and after the break. The QLR statistic is defined as the supremum Wald test statistic, allowing the break date *τ* to vary during the sample period. For improved performance, we use a symmetric trimming of 15%, where the first and last 15% of the observations are excluded from the estimation [53]. Additionally, we use both average and exponential Wald tests, where the former is defined as the average of the sample test statistic and the latter is the natural log of the average of the exponential of the sample test [54]. The break date is defined as the date when the maximum test statistic is reached.

### 3.4 Estimation issues

As with many time series analyses, autocorrelation and the appropriate selection of goodness-of-fit statistics can pose challenges. In the presence of autocorrelation, although the Ordinary Least Squares (OLS) estimator remains unbiased and consistent, statistical inferences may be affected due to unreliable standard errors, thus compromising the efficiency of the estimators [55]. A common diagnostic tool for detecting autocorrelation is the Durbin-Watson [56, 57] test. To address autocorrelation, two primary strategies are employed in the literature. The first involves adjusting the standard errors of the estimated parameters using the Newey-West [58] variance estimator. Alternatively, feasible generalized least squares (FGLS) can be applied to obtain more efficient estimators, with the Prais-Winsten and Cochrane-Orcutt techniques being notable procedures. Further details on these two approaches as well as the Durbin-Watson test are provided in Appendix A in S1 Appendix.

An additional concern is the presence of autocorrelation when utilizing the Box-Cox transformation. As previously discussed, the primary goal of this transformation is to normalize regression residuals and mitigate heteroskedasticity. However, as Savin and White [59] highlight, neglecting autocorrelation in the context of a Box-Cox transformation could result in misleading conclusions due to the potential presence of functional form misspecification. To ensure robustness, we implement the method proposed by Savin and White [59], which was later extended by Seaks and Layson [60]. The full details of these procedures are outlined in Appendix B in S1 Appendix.

Another important consideration is the assessment of model goodness-of-fit. When applying the FGLS method to correct for autocorrelation, the *R*^2^ statistic—which measures the proportion of variance in the dependent variable explained by the regressors—may not be directly comparable across models, as the dependent variable is transformed based on the autocorrelation coefficient estimated with different sets of regressors [55]. Similarly, when using the OLS coefficients with Newey-West errors, the traditional *R*^2^ maybe biased, since the variance formula for the dependent variable is no longer valid in the presence of a trend. Instead of the *R*^2^, we report stationarity statistics to evaluate whether the residuals exhibit stable statistical properties over time—a crucial assumption for ensuring that the error terms are *iid*. For this purpose, the Phillips-Perron test is employed to evaluate stationarity. Additionally, we report the Alkaike Information Criteria, a model selection metric that estimates the relative amount of information loss by a given model, as described in Banks and Joyner [61] for OLS regressions.

### 3.5 Data

Data on area and production are collected for 144 food commodities, including grains, oilseeds, fruits, fibers, and other crops, for 1961–2021 from FAOSTAT. These commodities combined account for over 98 percent of the global total agricultural land use during the sample period. The calorific information of most commodities is obtained from the FAO, as reported in the Food Balance Sheets. Appendix C in S1 Appendix details the commodities considered along with their calorific content.

Although many different data sources exist for commodity production and their nutritional values, considerable variation exists in how these data are collected (e.g., dry vs. raw weight). For consistency, we draw all data used in the analysis from FAO. In addition to the crop-level data, we also collect the regional-level data for each crop. Important to note that the FAO Food Balance Sheets provide the calorific content of a specific commodity per 100 grams of edible portion in terms of the retail weight ("as purchased"). As such, it does not consider the calories from the non-edible portion of the crop that can indirectly affect the food supply. For instance, some of the non-edible components, upon further processing, may be used as feed for livestock, which is an important source of calories for human consumption. Meanwhile, the FAOSTAT production data report the total production quantity of the crop, which includes both edible and non-edible components of the crop. In the present study, we assume that the relative ratio of edible and non-edible components for a given crop remains similar over the years. As such, the indirect contribution of the calorific content from non-edible should not severely bias the results.

## 4. Results

### 4.1 Production and yield patterns

**Fig 1:**
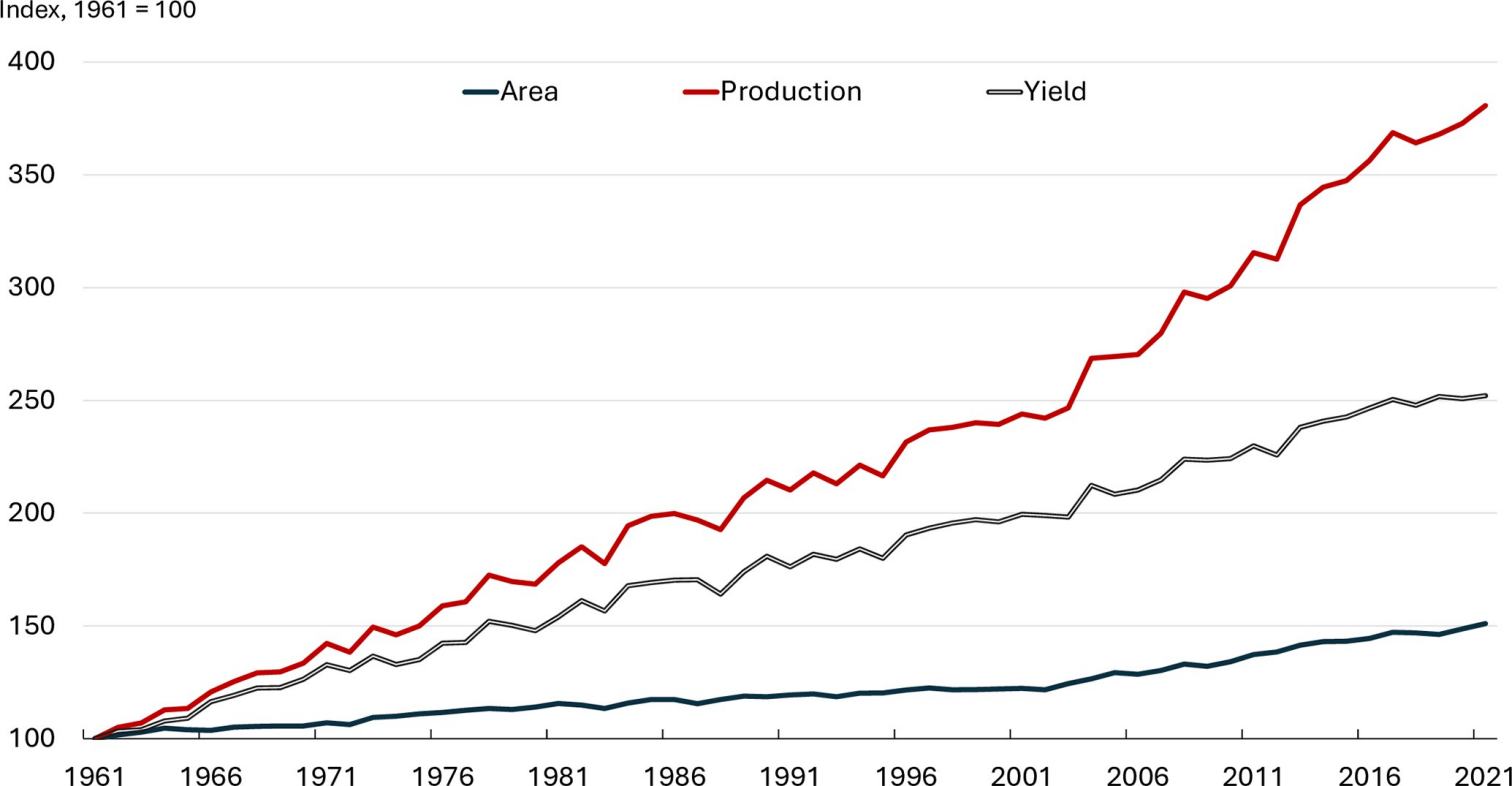
Global area, yield, and production. **Source:** Authors’ calculations based on FAO data.

**Fig 2:**
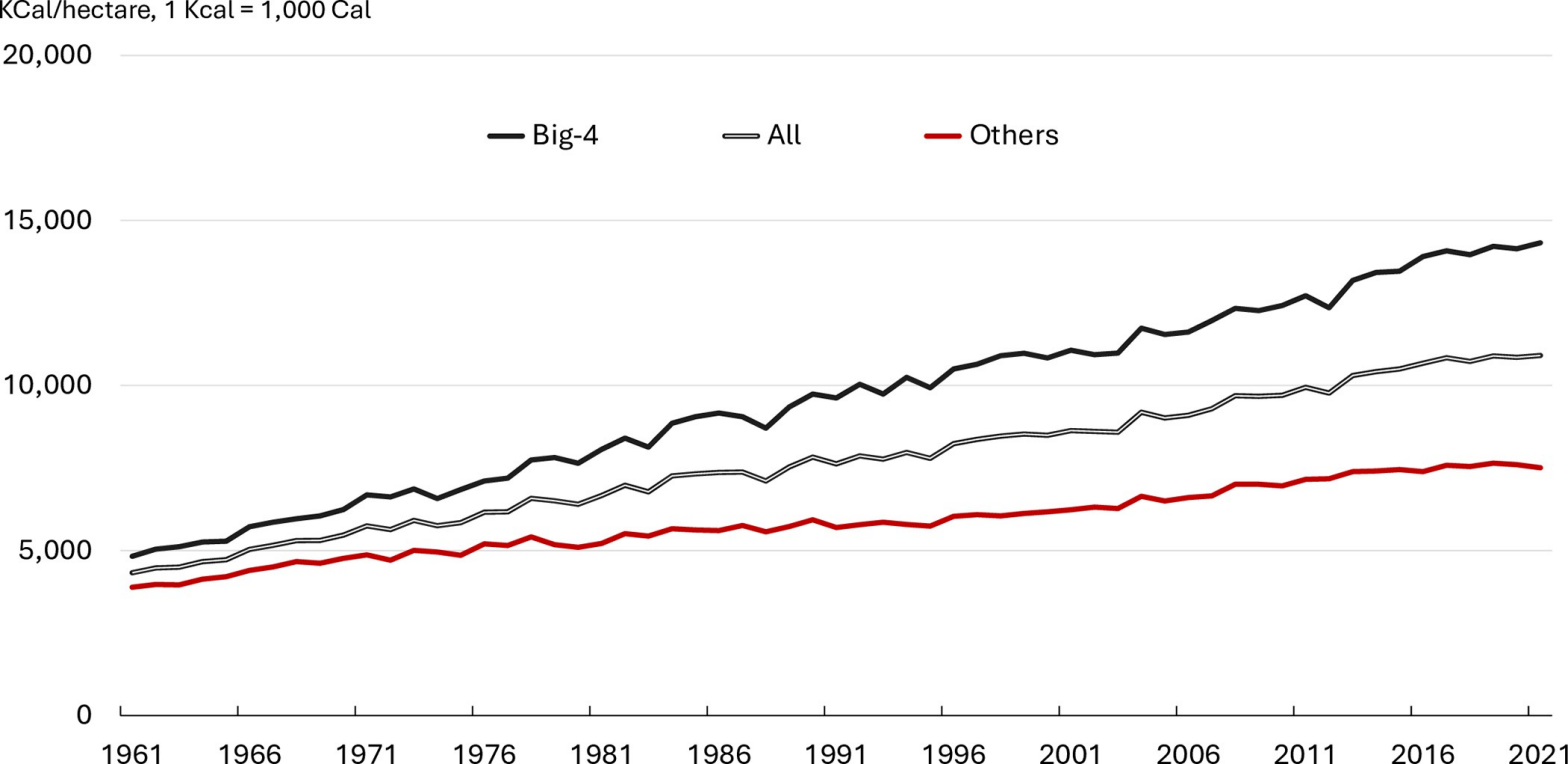
Yield growth by commodity grouping. **Source:** Authors’ calculations based on FAO data. **Notes:** Big-4 refers to the sum of maize, wheat, rice, and soybeans.

Fig 1 plots the global area, calorific production, and calorific yield across the 144 crops over the sample period, indexed to 100 in 1961. Global crop production nearly quadrupled over the past six decades on a calorific basis. Most of the increase reflects yield advancements, with cropland expansion experiencing much smaller growth. Fig 2 shows that the aggregate global yield has risen from approximately 4,330 *KCal/ha* in 1961 to almost 11,000 *KCal/ha* in 2021. Such growth, amounting to a 158% increase, closely mirrors the 152% increase in the global population over the same period.

Fig 2 further shows that much of the yield growth was driven by the Big-4 commodities (maize, wheat, rice, and soybeans), whose yields almost tripled from 4,826 *KCal/ha* in 1961 to 14,323 *KCal/ha* to 2021. In contrast, the yield of the “other” category (which includes the remaining 140 crops) grew by less than 100% over the sample period, with their 2021 calorific yield only half that of the Big-4.

**Fig 3:**
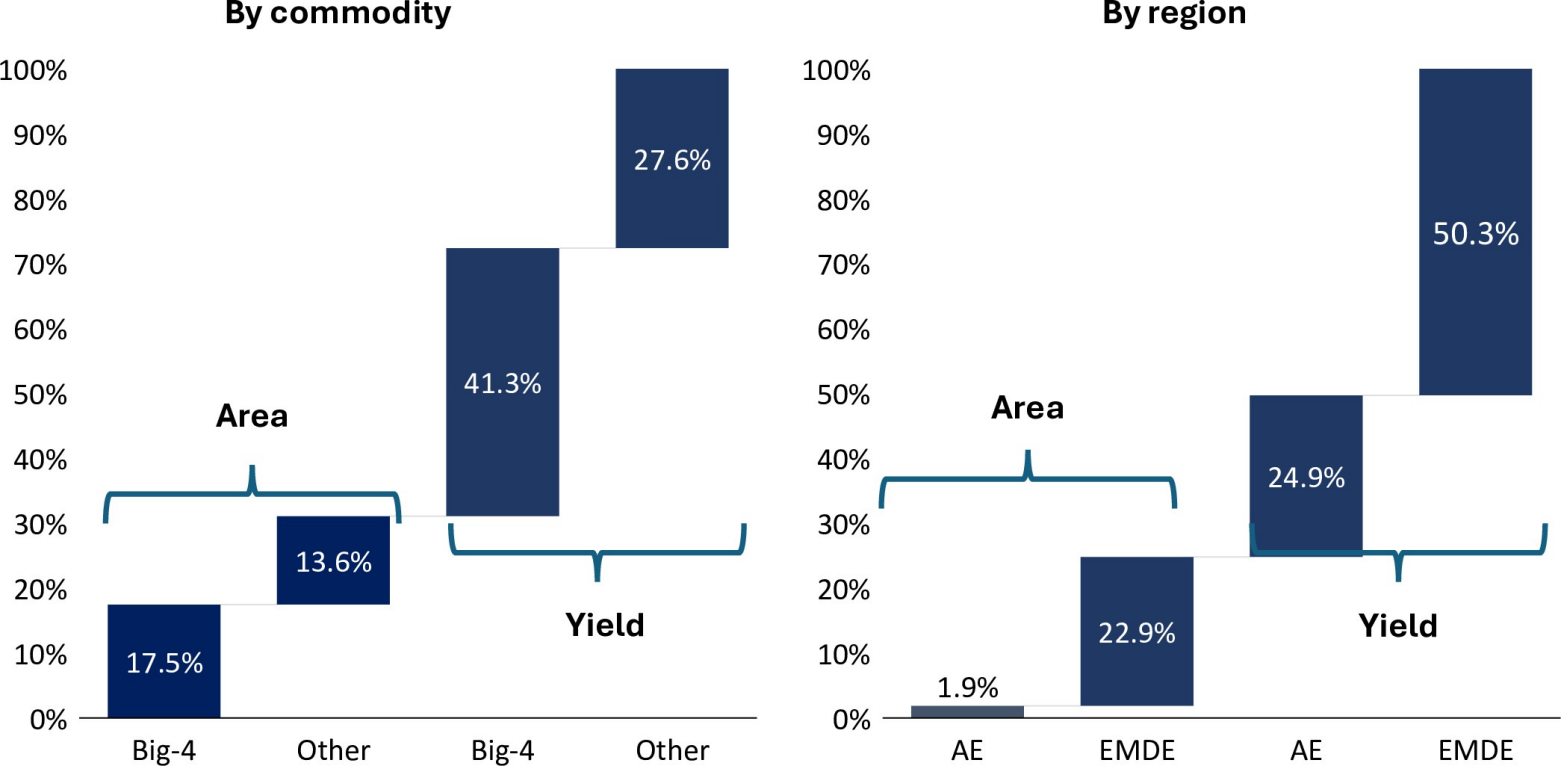
Area and yield contribution to production growth. **Source:** Authors’ calculations based on FAO data. **Notes:** Big-4 refers to the sum of maize, wheat, rice, and soybeans. Other includes all other commodities. ‘AE’ and ‘EMDE’ refer to Advanced Economies and Emerging Market and Development Economies, respectively.

Table 1 provides a more detailed decomposition of the area, yield, and production growth of the Big-4 versus other crops. We compute the average area, yield, and production for the first (1961–63, Panel A) and last (2019–21, Panel B) three years of the data, along with their respective shares. The middle panel shows their growth rates (calculated as logarithmic change) between the two periods, while the lower two panels present the contribution of area and yield to the total production growth. Fig 3 (left panel) summarizes the decomposition analysis based on Table 1.

**Table 1:** Decomposition of area, yield, and production growth by main crop.

	Maize	Wheat	Rice	Soybeans	Big-4	Other	All
**A. 1961–63 average**							
*Area (million hectares)*	105.8	206.0	118.3	24.0	454.1	517.5	971.6
*Yield (MCal/ha)*	7.1	3.8	5.4	3.8	5.0	3.9	4.4
*Production (million MCal)*	747.8	786.0	643.3	91.8	2,268.9	2,039.1	4,308.0
*Area (share*, *percent)*	10.9	21.2	12.2	2.5	46.7	53.3	100
*Production (share*, *percent)*	17.4	18.2	14.9	2.1	52.7	47.3	100
**B. 2016–19 average**							
*Area (million hectares)*	200.1	218.1	163.0	126.0	707.2	715.6	1,422.7
*Yield (MCal/ha)*	20.8	11.7	13.2	9.4	14.2	7.6	10.9
*Production (million MCal)*	4,166.2	2,551.6	2,155.8	1,187.0	10,060.6	5,427.5	15,488.2
*Area (share*, *percent)*	14.1	15.3	11.5	8.9	49.7	50.3	100.0
*Production (share*, *percent)*	26.9	16.5	13.9	7.7	65.0	35.0	100.0
**C. Growth from 1961–63 to 2019–21**							
*Area (log change)*	63.7	5.7	32.0	165.7	44.3	32.4	38.1
*Yield (log change)*	108.1	112.0	89.0	90.2	104.7	65.5	89.8
*Production (log change)*	171.8	117.7	120.9	256.0	148.9	97.9	128.0
**D. Contribution to growth from 1961–63 to 2019–21**							
*Area (percent)*	37.1	4.9	26.5	64.7	29.7	33.1	29.8
*Yield (percent)*	62.9	95.2	73.6	35.2	70.3	66.9	70.2
*Production (percent)*	100	100	100	100	100	100	100
**E. Contribution to growth from 1961–63 to 2019–21 (production-adjusted share)**							
*Area (percent)*	8.2	0.8	3.8	3.2	17.5	13.6	29.8
*Yield (percent)*	13.9	16.5	10.6	1.7	41.3	27.6	70.2
*Production (percent)*	22.1	17.4	14.4	4.9	58.8	41.2	100

**Notes:** “All” includes 144 commodities. “Big-4” is the sum of maize, wheat, rice, and soybeans. “Other includes” the remaining 140 commodities. Yield is measured as Mcal/hectare. The changes from 1961–63 to 2019–21 are calculated as logarithmic changes. The contribution of growth in panel E is evaluated at the average production shares of the two sub-periods. For example, the contribution of maize area is calculated as (all numbers are taken from the first column): 8.2 = 0.221*37.1, where 0.221 = 0.5*(0.174 + 0.269). Numbers may not add up due to rounding. **Source**: Authors’ calculations based on FAO data.

Three key findings emerge from the decomposition analysis. Firstly, more than two-thirds of global food production growth over the past six decades came from yield increases, while the remainder reflects area expansion. Secondly, the Big-4 commodities contributed nearly 60 percent to production growth with the rest of the growth attributable to all other crops. Thirdly, about two-thirds of the 60 percent production growth of the Big-4 is associated with yield increases.

**Fig 4:**
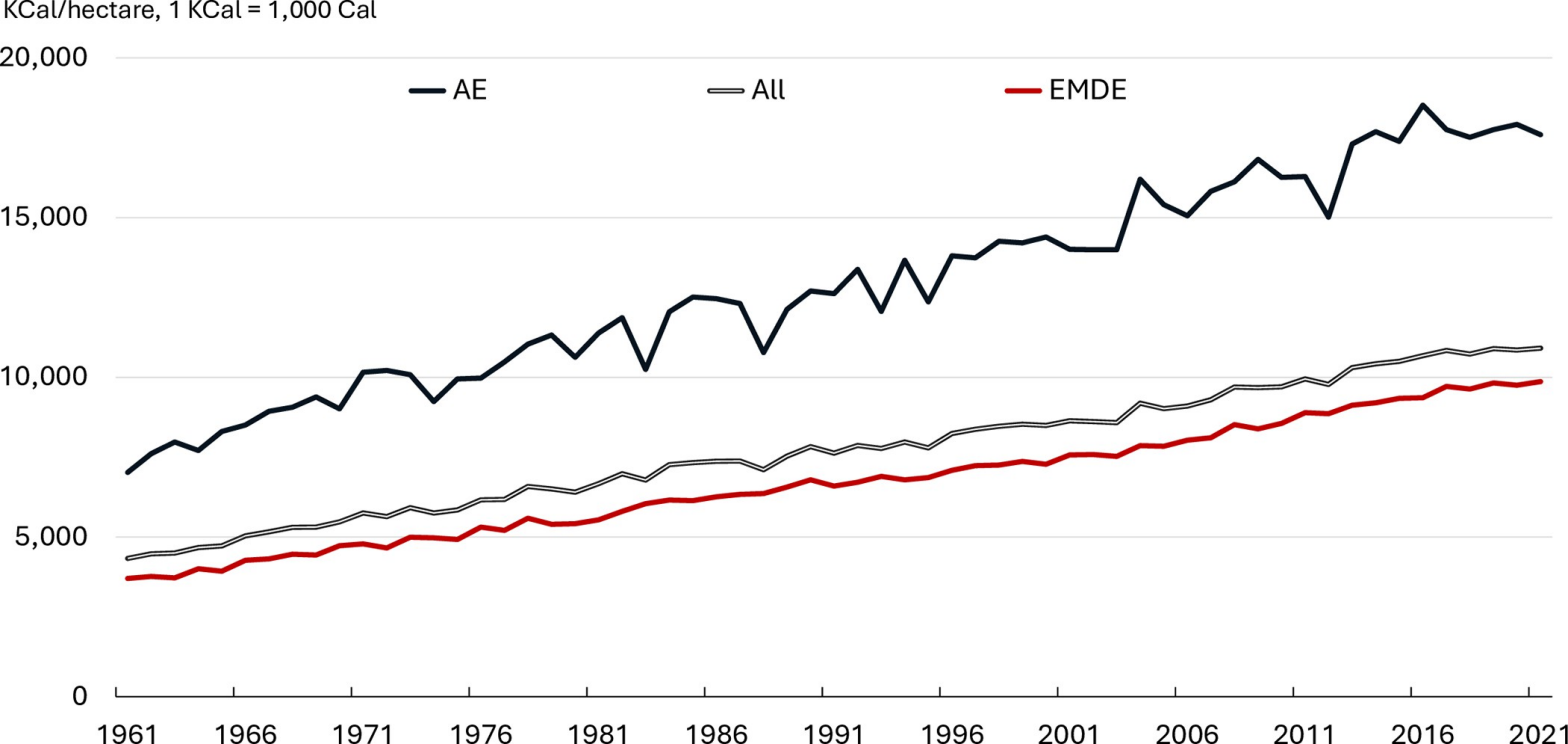
Yield growth by country grouping. **Source:** Authors’ calculations based on FAO data. **Notes:** ‘AE’ and ‘EMDE’ refer to Advanced Economies and Emerging Market and Development Economies, respectively.

Fig 4 shows a similar decomposition based on income and regional classification. First, it separates the country sample into (i) AEs (Advanced Economies) consisting primarily of countries in North America, Northern Europe, Western Europe, and Southern Europe and (ii) EMDEs (Emerging Markets and Developing Economies). Second, the EMDEs group is decomposed into five regional aggregates: LAC (Latin America and the Caribbean), SEAO (South-East Asia and Oceania), EECA (Eastern Europe and Central Asia), MENA (Middle East and North Africa), SSA (Sub-Saharan Africa). EMDEs have consistently reported lower aggregate yield than AEs. For example, the aggregate yield of EMDEs for 2021 was 60 percent lower than the corresponding yield of AEs. However, EMDEs’ yield growth slightly outpaced that of AEs—the former increased by 167% while the latter increased by 150% during the sample period.

Table 2 presents a detailed decomposition analysis of yield, area, and production for regional aggregates, which is further summarized in Fig 3 (right panel). Several results emerge from the regional decomposition analysis. First, during the past six decades, EMDEs contributed more than 70 percent to global production growth. Second, SEAO contributed nearly 40 percent of production growth, likely due to the Green Revolution (mostly in rice and less so wheat) which significantly enhanced productivity. Additionally, yield growth in EMDEs contributed to over 50% of global calorific production growth. In contrast, AEs contributed approximately a quarter of global calorific production growth through yield advancements. It is important to note that the latter witnessed substantial yield growth earlier in the 20th century, particularly following the introduction of hybrid crop varieties in the United States during the early 1930s [62].

**Table 2:** Decomposition of area, yield, and production growth by broad region.

	EECA	SEAO	SSA	MENA	LAC	EMDE	AE	All
**A. 1961–63 average**								
*Area (million hectares)*	188.4	397.5	88.8	43.4	73.3	791.3	180.2	971.6
*Yield (MCal/ha)*	3.9	3.7	3.0	3.4	4.5	3.7	7.5	4.4
*Production (million MCal)*	726.4	1,484.4	264.6	148.5	326.7	2,950.6	1,357.3	4,308.0
*Area (share*, *percent)*	19.4	40.9	9.1	4.5	7.5	81.4	18.6	100
*Production (share*, *percent)*	16.9	34.5	6.1	3.4	7.6	68.5	31.5	100
**B. 2019–21 average**								
*Area (million hectares)*	149.5	592.1	245.5	71.9	171.2	1,230.2	192.6	1,422.7
*Yield (MCal/ha)*	10.1	11.3	5.1	6.7	12.5	9.8	17.8	10.9
*Production (million MCal)*	1,513.9	6,688.4	1,241.1	482.7	2,143.3	12,069.4	3,418.8	15,488.2
*Area (share*, *percent)*	10.5	41.6	17.3	5.1	12.0	86.5	13.5	100.0
*Production (share*, *percent)*	9.8	43.2	8.0	3.1	13.8	77.9	22.1	100.0
**C. Growth from 1961–63 to 2019–21**								
*Area (log change)*	-23.2	39.9	101.8	50.5	84.8	44.1	6.6	38.1
*Yield (log change)*	96.5	110.7	52.8	67.6	103.3	96.8	85.7	89.8
*Production (log change)*	73.4	150.5	154.5	117.9	188.1	140.9	92.4	128.0
**D. Contribution to growth from 1961–63 to 2019–21**								
*Area (percent)*	-31.5	26.5	65.8	42.9	45.1	31.3	7.2	29.8
*Yield (percent)*	131.4	73.5	34.1	57.3	54.9	68.7	92.8	70.2
*Production (percent)*	100	100	100	100	100	100	100	100
**E. Contribution to growth from 1961–63 to 2019–21 (production-adjusted share)**								
*Area (percent)*	-4.2	10.3	4.7	1.4	4.8	22.9	1.9	29.8
*Yield (percent)*	17.5	28.5	2.4	1.9	5.9	50.3	24.9	70.2
*Production (percent)*	13.3	38.8	7.1	3.3	10.7	73.2	26.8	100

**Notes:** Yield is measured as MCal per hectare. The changes from 1961–63 to 2019–21 are calculated as logarithmic changes. Some numbers may not add up due to rounding. Acronyms denote regions according to the FAO classification: LAC (Latin America and the Caribbean), SEAO (South and East Asia, and Oceania), EECA (Eastern Europe and Central Asia), MENA (Middle East and North Africa), SSA (Sub-Saharan Africa). EMDE (Emerging Markets and Developing Economies) denotes the sum of the five preceding regions. AE (Advanced Economies) includes North America, Northern Europe, Western Europe, and Southern Europe. All refers to all regions. **Source**: Authors’ calculations based on FAO data.

### 4.2 Yield growth patterns

Our empirical analysis unfolds in the following steps. First, we test for structural breaks in the aggregate calorific yield indexes, applying both linear and exponential growth models. We then employ the Box-Cox transformation to identify which specification—linear or logarithmic yield indexes—best captures the yield growth patterns, considering models with and without a structural break or quadratic term. Based on the preferred transformation parameter we estimate the aggregate, commodity- and region-specific yield trajectories using Eqs (2), (4), and (5).

Results of the structural break analysis for the aggregate yield index are reported in Table 3. Under the linear transformation, the aggregate yield index appears to be subjected to a structural break in 1993, as suggested by the supremum, average, and exponential Wald tests, all of which are significant at the 1% level. For the logarithmic transformation, although the supremum Wald test suggests the presence of a structural break in 1978, the other two tests fail to reject the null hypothesis of no parameter shifts.

**Table 3:** Identification of structural break, aggregate yield index.

Wald test statistics	*y*	log(*y*)
*Supremum*	18.85***	162.39***
*Average*	8.38***	92.07
*Exponential*	6.94***	78.49
*Break year*	1993	1978

**Note:** Null hypothesis is that no structural break exists in yield (*y*) and logarithmic yield (log(*y*)) index.

The results also confirm that the optimal transformation parameter is close to one for linear model, with quadratic trend and break year of 1993. Indeed, statistics reported in Table 4 (last two columns) favor rejection of the null hypothesis that the optimal transformation parameter is equal to zero (logarithmic transformation) but fails to reject the null hypothesis that the parameter equals one (linear transformation). In Appendix B in S1 Appendix, we further consider the Box-Cox method in the presence of autocorrelation, finding that again a linear transformation is preferred over a logarithmic transformation for almost all models considered. Overall, Tables 3 and 4 suggest that a linear specification best captures global aggregate yield growth, which is consistent with some previous single-commodity studies [4, 63].

**Table 4:** Box-Cox transformation.

Regressors	*λ*	Null hypothesis	
		*λ* = 0	*λ* = 1
*Trend*	0.93 [0.77, 1.11]	68.29***	0.49
*Trend*, *trend*^*2*^	0.95 [0.39, 1.51]	12.60***	0.03
*Trend_before_1993*, *trend_after_1993*	1.07 [0.65, 1.48]	25.35***	0.10
*Trend_before_1978*, *trend_after_1978*	0.33 [-0.20, 0.68]	3.18*	13.73***

**Notes:** All regressions include a constant term. Square brackets (second column) denote confidence intervals of the optimal transformation parameter, *λ*, at a 95% significance level. The last three columns report test statistics of the null hypothesis of the logarithmic (*λ* = 0) and linear (*λ* = 1) transformations. Asterisks denote significance at 10 (*), 5 (**), and 1 (***) percent levels.

Based on the findings reported in Tables 3 and 4, we conduct regression analyses using the linear specification for the global aggregate as well as income and regional aggregates, with the results summarized in Tables 5–7. In each table, panel A reports parameter estimates of the base model, panel B shows the corresponding estimates which include a time-squared term, and panel C presents the results allowing for a structural break using piecewise linear regression. Before discussing the results, several key points should be noted. First, we test for residual autocorrelation in each regression using the Durbin-Watson test. Results in Table A1 in Appendix A in S1 Appendix suggest the presence of residual autocorrelation in almost all models. To address this, we use the Newey-West standard errors instead of the conventional standard errors in Tables 5–7.

**Table 5:** Parameter estimates for aggregate yield indices.

	All	Big-4	Other	AE	EMDE
**A. Base model**					
*Constant*	4332.75*** (41.08)	4691.98*** (47.48)	4020.06*** (50.27)	7401.90*** (131.02)	3526.23*** (34.60)
*Trend*	108.98*** (1.33)	158.05*** (1.62)	59.12*** (1.41)	175.71*** (4.20)	102.05*** (1.43)
*PP test*	-5.47***	-6.14***	-3.81***	-7.33***	-4.28***
*AIC*	803.07	836.07	795.83	955.25	796.18
**B. With Trend** ^**2**^					
*Constant*	4367.41*** (68.35)	4705.00*** (55.38)	4077.56*** (97.15)	7548.26*** (196.21)	3688.04*** (59.54)
*Trend*	105.67*** (5.65)	156.81*** (5.85)	53.64*** (6.85)	161.77*** (16.22)	86.64*** (5.00)
*Trend*^2^	0.05 (0.09)	0.02 (0.10)	0.09 (0.10)	0.22 (0.27)	0.25*** (0.08)
*P PP test*	-5.52***	-6.15***	-3.93***	-7.49***	-5.01***
*AIC*	804.60	838.03	796.37	956.56	785.64
**C. With a structural break in 1993**					
*Constant*	4366.77*** (55.31)	4679.28*** (45.70)	4091.72*** (82.58)	7574.11*** (166.17)	3640.02*** (41.18)
*Trend_before*	106.85*** (2.80)	158.84*** (2.82)	54.64*** (3.60)	164.95*** (8.07)	94.94*** (2.41)
*Trend_after*	4.47 (5.40)	-1.67 (6.47)	9.42 (5.85)	22.64 (16.58)	14.96*** (4.77)
*PP test*	-5.54***	-6.15***	-4.07***	-7.67***	-4.93***
*AIC*	804.24	838.00	793.57	955.48	786.86

**Notes**: Yield is measured as KCal per hectare. “All” includes 144 commodities. “Big-4” is the sum of maize, wheat, rice, and soybeans. “Other” includes the remaining 140 commodities. AE (Advanced Economies) includes North America, Northern Europe, Western Europe, and Southern Europe; EMDE (Emerging Markets and Developing Economies) includes LAC, SEAO, EECA, and MENA regions based on FAO classifications. Numbers in parenthesis refer to Newey-West standard errors that account for heteroskedasticity and autocorrelation. PP test refers to the Philips-Perron stationarity test, with the null hypothesis that the series (here residuals) under consideration contains a unit root. AIC refers to Akaike Information Criteria. Asterisks denote significance at 10 (*), 5 (**), and 1 (***) percent levels.

**Table 6:** Parameter estimates for commodity group-based yield indices.

	Cereals & grains	Oil crops	Fruits & vegetables	Other crop group
**A. Base model**				
*Constant*	4209.09*** (58.13)	2367.97*** (113.48)	2949.48*** (45.57)	5738.48*** (137.43)
*Trend*	151.98*** (2.39)	105.78*** (3.24)	37.12*** (1.43)	49.20*** (3.74)
*PP test*	-4.74***	-2.70**	-1.34	-2.01
*AIC*	854.71	868.55	801.37	862.69
**B. With Trend** ^**2**^				
*Constant*	4496.18*** (73.31)	2964.65*** (42.00)	3204.01*** (83.77)	5229.09*** (120.48)
*Trend*	124.64*** (6.73)	48.96*** (4.85)	12.88** (5.98)	97.71*** (7.73)
*Trend* ^2^	0.44*** (0.11)	0.92*** (0.10)	0.39*** (0.09)	-0.78*** (0.11)
*PP test*	-5.78***	-6.61***	-2.86**	-3.98***
*AIC*	841.24	780.66	769.92	805.91
**C. With a structural break in 1993**				
*Constant*	4412.13*** (49.79)	2809.76*** (40.33)	3170.69*** (62.62)	5418.00*** (134.00)
*Trend_before*	139.29*** (3.14)	78.17*** (1.87)	23.29*** (2.62)	69.23*** (5.51)
*Trend_after*	26.69*** (6.91)	58.08*** (4.82)	29.08*** (4.10)	-42.13*** (8.89)
*PP test*	-5.67***	-6.94***	-3.29**	-3.12**
*AIC*	842.61	778.50	749.35	828.09

**Notes:** Yield indices for each group of commodities are measured in KCal per hectare. “Other crop groups” includes pulses, roots and tubers, and treenuts; in other words, this category includes all other crops excluding cereals & grains, oil crops, and fruits & vegetables. Numbers in parenthesis refer to Newey-West standard errors that account for heteroskedasticity and autocorrelation. PP test refers to the Philips-Perron stationarity test, with the null hypothesis that the series (here residuals) under consideration contains a unit root. AIC refers to Akaike Information Criteria. Asterisks denote significance at 10 (*), 5 (**), and 1 (***) percent levels.

**Table 7:** Parameter estimates for region-based yield indices.

	EMDE	LAC	SEAO	EECA	MENA	SSA
**A. Base model**						
*Constant*	3526.23*** (34.60)	3383.13*** (201.58)	3359.53*** (42.17)	4097.24*** (183.06)	3138.13*** (67.30)	2807.17*** (43.00)
*Trend*	102.05*** (1.43)	139.38*** (6.00)	131.20*** (1.36)	84.97*** (6.43)	65.13*** (2.36)	35.61*** (1.08)
*PP test*	-4.28***	-2.85**	-3.45**	-4.27***	-7.77***	-5.34***
*AIC*	796.18	945.92	803.07	977.69	867.87	786.63
**B. With Trend** ^**2**^						
*Constant*	3688.04*** (59.54)	4511.85*** (68.38)	3420.02*** (48.75)	4713.93*** (371.94)	3177.91*** (99.75)	2886.82*** (51.63)
*Trend*	86.64*** (5.00)	31.88*** (7.23)	125.44*** (5.13)	26.24 (27.00)	61.34*** (8.66)	28.02*** (4.12)
*Trend* ^2^	0.25*** (0.08)	1.73*** (0.13)	0.09 (0.08)	0.95** (0.40)	0.06 (0.15)	0.12* (0.07)
*PP test*	-5.01***	-8.52***	-3.46**	-4.92***	-7.82***	-5.53***
*AIC*	785.64	856.65	803.64	970.65	869.66	785.33
**C. With a structural break in 1993**						
*Constant*	3640.02*** (41.18)	4221.02*** (51.03)	3363.30*** (49.43)	4571.64*** (283.60)	3188.07*** (77.70)	2873.56*** (51.04)
*Trend_before*	94.94*** (2.41)	87.01*** (2.91)	130.96*** (2.78)	55.32*** (13.67)	62.01*** (3.90)	31.46*** (2.26)
*Trend_after*	14.96*** (4.77)	110.15*** (6.55)	0.50 (5.32)	62.37** (24.70)	6.56 (9.21)	8.73** (4.22)
*PP test*	-4.93***	-9.04***	-3.45**	-4.95***	-7.87***	-5.59***
*AIC*	786.86	853.27	805.06	969.77	869.25	784.37

**Notes:** Yield is measured as KCal per hectare. Definitions of regional classification can be found in Table 2. Numbers in parenthesis refer to Newey-West standard errors that account for heteroskedasticity and autocorrelation. PP test refers to the Philips-Perron stationarity test, with the null hypothesis that the series (here residuals) under consideration contains a unit root. AIC refers to Akaike Information Criteria. Asterisks denote significance at 10 (*), 5 (**), and 1 (***) percent levels.

Second, we re-estimated the models using the Prais-Winsten FGLS estimator, which directly accounts for autocorrelation, rather than simply adjusting the standard errors as in the Newey-West approach. The results, reported in Appendix A (Tables A2-A4 in S1 Appendix), indicate that most of the statistical significance remains consistent and the estimated coefficients are closely aligned with those in Tables 5–7. Third, as the Phillips-Perron stationarity statistics confirm, most of the residuals do not contain a unit root, suggesting that the statistical properties of the residuals generally do not change over time.

Lastly, the results from AIC vary depending on the yield indices analyzed. In Table 5, the base model with a linear trend exhibits the lowest AIC for the aggregate, Big-4, and AE yield indices. In contrast, for Other and EMDE yield indices, the models with a structural break and a quadratic trend, respectively, give lower AIC values. In Tables 6 and 7, the quadratic trend model is preferred for cereals & grains and other crop group, the linear trend model for MENA and SEAO, and the structural break model for all other commodity and regional groupings.

The first column of Table 5 focuses on the aggregate global yield index. As can be seen, the parameter estimate for the post-1993 term is non-significant. Similarly, the parameter estimate for the quadratic trend was positive but non-significant. Combined, these findings suggest that aggregate global calorific yield has exhibited steady growth at approximately 109 KCal/ha per year with no evidence of deceleration or acceleration.

At a more granular level, the second and third columns of Table 5 show that the yield growth of the Big-4 outpaced other crops by a considerable margin, with the gap between the two groups widening over time, consistent with Fig 2. The average yield of Big-4 commodities grew at 158 *KCal* per year during the sample period, with no evidence of yield growth acceleration or deceleration. By contrast, the yield of ‘Other’ grew only at 59 *KCal* per year over the period of analysis. While the parameter estimate of the quadratic term is non-significant, the model with structural break suggests that the yield of the “other” crop aggregates accelerated from 55 *KCal* before 1993 to 64 *KCal* per year after 1993.

The last two columns in Table 5 show the estimation results for AE and EMDE country groupings. On average, the aggregate yield in the AEs grew by 76 *KCal/ha* per year, while in EMDEs it grew by 95 and 109 *KCal/ha* before and after 1993, respectively. The acceleration in EMDEs was likely driven by productivity growth in Latin America (following the uptake of soybean production), efficiency gains in Eastern Europe and Central Asia (following the collapse of centrally planned economies), and Asia (following the adoption of the green revolution). In contrast, Sub-Saharan Africa did not contribute much to the acceleration due to its low crop intensity, inadequate irrigation systems, and limited use of commercial inputs during the sample period [6].

Further disaggregation at commodity and regional levels is reported in Tables 6 and 7. Specifically, Table 6 reports yield growth estimates for four commodity groups: cereals and grains, oil crops, fruits and vegetables, and other crops. Non-linearities are present in all four groups, as demonstrated by the significant quadratic and post-1993 coefficient estimates. Consistent with the studies on individual commodities discussed earlier, regression analyses by commodity groups gave a mixed picture: yield growth accelerated in cereals, oil crops, and fruits/vegetables, and decelerated in the remainder crop aggregates (the “others” category in the table). Cereals and grains present the highest yield growth during the sample period, as illustrated by the large magnitude of the coefficient associated with the linear trend. However, the magnitude of the quadratic term, as well as the estimated parameter after the structural break in 1993, are considerably larger for oil crops, suggesting that the yield growth for these crops may eventually catch up with cereals and grains.

Table 7 reports parameter estimates for regional aggregates. Results show that yield growth acceleration, although with a somewhat small magnitude, is present in EMDEs, mainly reflecting the growth in LAC and EECA. For SEAO, MENA, and SSA, neither the quadratic term nor the post-structural break parameter is statistically significant, suggesting linear yield growth in these regions. However, yield growth in SEAO has been the fastest across all EMDEs (although without acceleration) during the sample period.

## 5. Discussion and conclusion

Understanding yield growth patterns is crucial for the discussion of food security and sustainable development. However, the frameworks employed by existing studies are inadequate for assessing yield growth aggregated across all food commodities and countries. In this study, we introduce an innovative calorific-based approach to examine aggregate global crop yield growth. Our analyses indicate that, contrary to the widely accepted view, the global aggregate crop yield has grown at a consistent rate of 109 KCal/ha per year from 1961 to 2021 (equivalent to adding 32.6 kilograms of wheat every year in each hectare of land), with no evidence of deceleration or acceleration. This suggests that yield stagnation or growth deceleration for specific commodities, regions, or countriesdiscussed in the literature has been offset by acceleration elsewhere, resulting in a broadly stable aggregate yield growth pattern.

This calorific-based approach further allows us to examine the yield growth at various commodity groups or regional levels in a consistent manner. We show that, while the yield of the Big-4 commodities exhibited no acceleration or deceleration, the aggregates of the remaining crops showed a slight acceleration after 1993. Similarly, while yield growth in AEs has been fairly constant, EMDEs experienced some acceleration, driven primarily by three regions (LAC, EECA, and SSA). Combined, these findings highlight the importance of analyzing aggregate trends based on the single metrics developed in this paper alongside the performance of specific commodities, countries, or regions reported in the literature.

Yield growth during the past six decades has supported adequate food supplies at a global level. However, several challenges must be addressed to ensure that future yield growth patterns meet global food requirements, which are expected to grow by one-third over the next three decades due to population and income growth as well as changing consumption patterns [64, 65].

The first challenge is the increasing frequency and intensity of adverse weather patterns, exacerbated by the ongoing climate change, which is expected to alter the regional composition of commodity production and increase yield volatility [66]. Public investment in developing new cultivars resilient to temperature and precipitation variations will be crucial for maintaining historical yield growth trajectories. The significance of such cultivars will only grow, as most global food supplies rely heavily on a few crops, with the Big-4 accounting for nearly half of the global calorie supplies [67]. Additionally, climate change is likely to increase yield volatility. Indeed, yield growth in advanced economies has been more volatile compared to emerging markets and developing economies, primarily due to weather-induced factors such as the La Niña episodes of 2010–11 and 2011–12 [68]. Addressing these challenges will require substantial investment, research and development, and diffusion efforts, similar to those that led to the development and use of hybrid maize varieties in the United States and the grain varieties promoted by the Consultative Group on International Agricultural Research, which spearheaded the Green Revolution in East Asia [10, 69, 70].

Another challenge is removing distorting trade policies, which restrict food availability in regions experiencing food deficits [71–73]. Despite agricultural policy reforms undertaken in recent decades, global agricultural trade is still subjected to trade barriers and domestic policies [74]. Policies diverting food commodities to biofuels should balance environmental and energy security concerns and food availability [75]. Likewise, policies to reduce greenhouse gas emissions should not compromise productivity. This is especially important since agriculture accounts for a quarter of global emissions [76].

Finally, even if adequate food supplies can be ensured globally, access to food remains a concern, especially in low-income countries where food insecurity is associated with conflict and extreme weather events [77]. The number of people subjected to acute food insecurity worldwide has more than doubled during the past five years, exceeding an estimated 280 million in 2023 [78]. Achieving equitable access to food across income groups will entail financial assistance, food delivery through aid, as well as investments and policies aimed at increasing productivity but also reducing food waste at the production level [26].

The calorific-based indices along with the estimation procedure presented in this paper not only deepen our comprehension at both aggregate and regional levels but also pave the way for multifaceted avenues of future research. For instance, by incorporating additional factors like water usage or chemical input use, the aggregation of production and yields could facilitate an assessment of environmental strain. Moreover, exploring alternative aggregation methods could elucidate the susceptibility of various commodities and regions to changes in weather patterns and intensification of climatic phenomena like El Niño. Lastly, our proposed index methodology holds promise in scrutinizing patterns of production or yield volatility, thereby offering valuable insights into the resilience of food production systems.

## Supporting information

S1 Appendix (PDF)
