# Supplementary material for: Yield growth patterns of food commodities: Insights and challenges
Source: PLoS One. 2024 Nov 27;19(11):e0313088. doi: 10.1371/journal.pone.0313088 (PMC11602064; doi:10.1371/journal.pone.0313088)
Supplement: S1 Appendix — (PDF) [file pone.0313088.s001.pdf]

Appendix A. Autocorrelation: Detection and Remedial Procedures

This appendix describes the standard methods for detecting and addressing autocorrelation in regression analyses.

A.1. Testing for Autocorrelation

Consider the following linear model:

$$y_t = \boldsymbol{X}_t\boldsymbol{\beta}_1 + u_t, \tag{A.1.1}$$

where y represents the dependent variable, $\boldsymbol{X_t}$ is the matrix of independent variables, $\boldsymbol{\beta_1}$ is the coefficient vector, u is the error term, and $t = 1,2,\dots,T$ denotes the time index. A common form of autocorrelation is the AutoRegressive process of order 1, or AR(1), where the error term follows:

$$u_t = \rho u_{t-1} + \eta_t, \tag{A.1.2}$$

with η_t being an independently and identically distributed (i.i.d.) random variable, $N(0, \sigma_\eta^2)$.

The Durbin-Watson (DW) test is a widely used method for detecting first-order autocorrelation. The test statistic is defined as (Durbin and Watson 1950, 1951):

$$d = \frac{\sum_{t=1}^{T-1}(\hat{u}_{t+1} - \hat{u}_t)^2}{\sum_{t=1}^{T}\hat{u}_t^2}, \tag{A.1.3}$$

where $\hat{u}_t$ denotes the estimated residual. The d statistic ranges from 0 and 4. Under the null hypothesis of no first-order autocorrelation (i.e., ρ=0), the value of d is approximately 2. Savin and White (1977) provide empirical upper and lower bounds for various sample sizes and number of regressors

To detect higher-order autocorrelations, Durbin's alternative test, the Lagrange Multiplier (LM) test, can beused. Further details of this approach are outlined found in Godfrey (1988).

Table A1 presents the DW test results for the regressions presented in Tables 5-7 in the paper.

Table A1. Durbin-Watson Autocorrelation test statistic for Models in Tables 5-7

	All	Big-4	Other	AE	EMDE	Cereals & grains	Oil crops	Fruits & veg	Other	LAC	SEAO	EECA	MENA	SSA
base	**1.32**	1.55	**0.79**	1.90	**0.95**	**1.10**	**0.40**	**0.18**	**0.35**	**0.49**	**0.66**	**0.98**	1.99	**1.29**
w. trend^2	**1.33**	1.55	**0.81**	1.92	**1.16**	**1.42**	1.73	**0.32**	**0.88**	2.22	**0.68**	1.14	2.00	**1.36**
w. break	**1.34**	1.56	**0.85**	1.96	**1.14**	**1.39**	1.78	**0.44**	**0.63**	2.31	**0.66**	1.15	2.02	**1.38**

Notes: The base model refers to the regression of $y = \beta_0 + \beta_1 trend + u$. The model with trend^2 refers to the regression of $y = \beta_0 + \beta_1 trend + \beta_2 trend^2 + u$. The model with break is $y = \beta_0 + \beta_1 trend\_before + \beta_2 trend\_after + u$, where the structural break occurs in 1993. The null hypothesis is that the regression model does not contain AR(1). Numbers in bold suggest the null hypothesis can be rejected for the corresponding regression model reported in Tables 5-7 in the paper.

The DW bounds for the base model (with trend only) are [1.55, 1.62] while for the other two models, the bounds are [1.51, 1.65]. The null hypothesis can be rejected if d falls below the lower bound. As shown in Table B1, of the 14 sets of models reported,only five (Big-4, AE, Oil Crops, LAC, and MENA) do not exhibit AR(1) autocorrelation.

A.2 Remedial procedures for autocorrelation

It is important to note that, even in the presence of autocorrelation, the conventional Ordinary Least Square (OLS) estimator remains *unbiased* and *consistent*, as long as the explanatory variables are exogenous (Wooldridge 2016). This holds regardless of the degree of autocorrelation, much like the case of heteroskedasticity in cross-sectional data. However, the OLS estimator is no longer the Best Linear Unbiased Estimator (BLUE), and the standard errors along with the test statistics are no longer valid.

To address autocorrelation, two common remedial approaches are outlined below:

1. Newy-West autocorrelation and heteroskedastic consistent standard errors.

Since the OLS estimator is unbiased and consistent, one solution is using the Newey-West (1987) heteroskedasticity and autocorrelation-consistent (HAC) standard errors. Considering the same model as in Equation (A.1.1), the HAC variance estimator for an autoregression of order m (where m>0) is given by:

$$\widehat{Var}\left(\hat{\beta}_{OLS}\right) = (\boldsymbol{X}'\boldsymbol{X})^{-1}\boldsymbol{X}'\widehat{\boldsymbol{\Omega}}\boldsymbol{X}(\boldsymbol{X}'\boldsymbol{X})^{-1}, \tag{A.2.1}$$

where $$\boldsymbol{X}'\widehat{\boldsymbol{\Omega}}\boldsymbol{X} = \frac{T}{T-k}\sum_{t=1}^{T}\hat{u}_t^2\boldsymbol{x}_t'\boldsymbol{x}_t + \frac{T}{T-k}\sum_{l=1}^{m}\left(1-\frac{l}{m+1}\right)\sum_{t=l+1}^{T}\hat{u}_t\hat{u}_{t-l}(\boldsymbol{x}_t'\boldsymbol{x}_{t-l} + \boldsymbol{x}_{t-l}'\boldsymbol{x}_t). \tag{A.2.2}$$

In this expression, k represents the number of regressors, $\boldsymbol{x_t}$ is the row of the X matrix observed at time t, $l = 1,2, \dots m$ is the autoregressive order, and $\widehat{\boldsymbol{\Omega}}$ denotes the variance-covariance matrix of the residuals.

Newey-West variance estimator adjusts for the autocorrelation in the error term, with the correlation decreasing as the time gap between error terms widens. Newey and West (1987) demonstrate that this estimator is consistent in the presence of both autocorrelation and heteroskedasticity.

2. Prais-Winsten feasible generalized linear squares estimator

An alternative approach to address autocorrelation is to use an efficient estimator that remains unbiased and consistent. Assuming the error follows an AR(1) process as in Equation (A.1.2), then the variance of u_t in Equation (A.1.1) can be expressed as $Var(u_t) = (\sigma_\eta^2)/(1-\rho^2)$. Cochrane and Orcutt (1949) recommend the following transformation for observations $t = 2, \dots, T$:

$$y_t - \rho y_{t-1} = \beta_0(1-\rho) + \boldsymbol{\beta_1}(\boldsymbol{X}_t - \rho X_{t-1}) + e_t, \tag{A.2.3}$$

where $e_t = u_t - \rho u_{t-1}$. Since $Var(e_t) = Var(u_t - \rho u_{t-1}) = Var(u_t) + \rho^2 Var(u_{t-1}) - 2\rho Cov(u_t, u_{t-1}) = \sigma_\eta^2$, regression in Equation (B.2.3) is not subject to autocorrelation. The OLS estimator based on this transformation is a feasible generalized least squares (FGLS) estimator, commonly known as the Cochrane-Orcutt procedure.

Prais and Winsten (1954) recommend to include the following equation for $t = 1$:

$$\sqrt{1-\rho^2}y_1 = \beta_0\sqrt{1-\rho^2} + \boldsymbol{\beta_1}\sqrt{1-\rho^2}\boldsymbol{x_1} + \sqrt{1-\rho^2}e_1 \tag{A.2.4}$$

The Prais-Winsten GLS estimator is BLUE, and because the error terms in both transformed equations are free from autocorrelation and potential heteroskedasticity, standard statistical inferences are valid.

Both the Newey-West HAC standard errors and the Prais-Winsten FGLS estimator effectively correct for autocorrelation in regression analysis. However, as Wooldridge (2016, p. 388, Chapter 12) notes:

> *"(i)n recent years, it has become popular to estimate models by OLS but to correct the standard errors for fairly arbitrary forms of serial correlation (and heteroscedasticity). Even though we know OLS will be inefficient, there are some good reasons for taking this approach. First, the explanatory variables may not be strictly exogenous. In this case, FGLS is not even consistent, let alone efficient. Second, in most applications of FGLS, the errors are assumed to follow an AR(1) model. It may be better to compute standard errors for the OLS estimates that are robust to more general forms of serial correlation."*

In eth main text presents we follow Wooldridge's (2016) recommendation by reporting the OLS estimator with standard errors corrected for serial correlation via Newey-West HAC estimators (see Tables 5-7). Notably, the use of HAC standard errors did not significantly alter the statistical significance of most parameter estimates compared to conventional standard errors.

For robustness, we also estimated the models using the Prais-Winsten FGLS estimator, assuming an AR(1) error process. These results are reported in Tables A2-A4 in this appendix. Additionally, we tested an AR(2) process, yielding results that are qualitatively similar to those reported in Tables B2-B4.

As shown in Tables A2-A4, the results align closely with those in the main text (Tables 5-7) in terms of statistical significance and coefficient magnitudes. Two key differences, highlighted in blue, include:

(1) **Fruits and Vegetables (with trend and trend-squared):** The Prais-Winsten FGLS estimator doubles the trend coefficient compared to OLS (24.54 vs. 12.88, both significant), while the trend-squared coefficient becomes non-significant.

(2) **SSA (with trend and trend-squared, and with structural break):** In the Prais-Winsten FGLS regression quadratic trend term and the post-1993 coefficient both become non-significant.

References:

Cochrane, Donald, and Guy H. Orcutt. "Application of least squares regression to relationships containing auto-correlated error terms." *Journal of the American Statistical Association* 44, no. 245 (1949): 32-61.

Durbin, J., and G. S. Watson. "Testing for Serial Correlation in Least Squares Regression: I." *Biometrika* 37, no. 3/4 (1950): 409–28. https://doi.org/10.2307/2332391.

Durbin, J., and G. S. Watson. "Testing for Serial Correlation in Least Squares Regression. II." *Biometrika* 38, no. 1/2 (1951): 159–77. https://doi.org/10.2307/2332325.

Godfrey, Leslie G. *Misspecification tests in econometrics: the Lagrange multiplier principle and other approaches*. No. 16. Cambridge University Press, 1988.

Newey, Whitney K., and Kenneth D. West. "A simple, positive semi-definite, heteroskedasticity and autocorrelation consistent covariance matrix." (1986).

Prais, S. J., and C. B. Winsten. "Trend estimators and serial correlation, Cowles Commission Monograph, no. 23." (1954).

Savin, Nathan E., and Kenneth J. White. "The Durbin-Watson test for serial correlation with extreme sample sizes or many regressors." *Econometrica: Journal of the Econometric Society* (1977): 1989-1996.

Wooldridge, J. M. "Introductory econometrics: A modern approach (6th,)." Mason, OH: South-Western (2016).

Table A2. Regression results with Prais-Winsten estimator assuming AR(1)

	All	Big-4	Other	AE	EMDE
**A. Base model**					
Constant	4330.15***	4691.61***	4007.90***	7401.13***	3527.37***
	(62.07)	(72.47)	(78.57)	(161.00)	(72.30)
Trend	109.01***	158.05***	59.28***	175.71***	102.12***
	(1.73)	(2.03)	(2.18)	(4.51)	(2.01)
Adj. R^2	0.98	0.99	0.91	0.96	0.97
PP test	-5.47***	-6.14***	-3.80***	-7.33***	-4.28***
**B. With $Trend^2$**					
Constant	4354.03***	4703.08***	4018.45***	7545.49***	3678.95***
	(93.03)	(110.56)	(113.77)	(242.56)	(86.51)
Trend	106.71***	156.95***	58.25***	161.99***	87.31***
	(6.93)	(8.23)	(8.47)	(18.05)	(6.44)
$Trend^2$	0.04	0.02	0.02	0.22	0.24**
	(0.11)	(0.13)	(0.13)	(0.28)	(0.10)
Adj. R^2	0.98	0.99	0.91	0.96	0.97
PP test	-5.51***	-6.15***	-3.83***	-7.49***	-5.01***
**C. With a structural break in 1993**					
Constant	4357.68***	4678.26***	4053.72***	7574.32***	3636.89***
	(80.17)	(95.56)	(94.98)	(202.56)	(77.27)
Trend_before	107.27***	158.89***	56.38***	164.94***	95.07***
	(3.69)	(4.39)	(4.39)	(9.29)	(3.56)
Trend_after	3.65	-1.77	6.06	22.66	14.76**
	(6.86)	(8.17)	(8.14)	(17.30)	(6.62)
Adj. R^2	0.98	0.99	0.92	0.97	0.98
PP test	-4.82***	-6.15***	-4.01***	-7.67***	-4.93***

Notes: Yield is measured as KCal per hectare. "All" includes 144 commodities. "Big-4" is the sum of maize, wheat, rice, and soybeans. "Other" includes the remaining 140 commodities. Standard errors are reported in parenthesis. AE (Advanced Economies) includes North America, Northern Europe, Western Europe, and Southern Europe; EMDE (Emerging Markets and Developing Economies) includes LAC, SEAO, EECA, and MENA regions based on FAO classifications. PP test refers to the Philips-Perron stationarity test, with the null hypothesis that the series (here residuals) under consideration contains a unit root. Numbers in parentheses are standard errors. Asterisks denote significance at 10 (*), 5 (**), and 1 (***) percent levels.

Table A3. Parameter estimates for commodity group-based yield indices with Prais-Winsten estimator assuming AR(1)

	Cereals & grains	Oil crops	Fruits & vegetables	Other
**A. Base model**				
Constant	4208.48***	2470.82***	2892.51***	5678.30***
	(106.76)	(189.74)	(155.27)	(218.72)
Trend	152.12***	103.66***	39.56***	48.29***
	(2.98)	(5.16)	(3.97)	(5.87)
Adj. R^2	0.97	0.84	0.73	0.66
PP test	-4.74***	-2.53	-1.40	-2.10
**B. With Trend²**				
Constant	4484.23***	2963.06***	3035.28***	5205.30***
	(120.33)	(60.28)	(133.90)	(119.61)
Trend	125.50***	49.17***	24.54**	100.10***
	(8.96)	(4.49)	(9.72)	(8.90)
$Trend^2$	0.43***	0.91***	0.23	-0.83***
	(0.14)	(0.07)	(0.15)	(0.14)
Adj. R^2	0.99	0.99	0.81	0.88
PP test	-5.78***	-6.61***	-2.08	-3.99***
**C. With a structural break in 1993**				
Constant	4406.14***	2810.47***	3087.59***	5367.65***
	(107.31)	(50.50)	(87.79)	(150.64)
Trend_before	139.55***	78.16***	26.79***	71.69***
	(4.94)	(2.32)	(4.06)	(6.97)
Trend_after	26.28***	58.08***	23.99***	-48.13***
	(9.18)	(4.82)	(7.47)	(12.89)
Adj. R^2	0.99	0.99	0.88	0.80
PP test	-5.67***	-6.94***	-2.82*	-3.13**

Notes: Yield indices for each group of commodities are measured in KCal per hectare. "Other" includes pulses, roots and tubers, and treenuts; in other words, this category includes all other crops excluding cereals & grains, oil crops, and fruits & vegetables. PP test refers to the Philips-Perron stationarity test, with the null hypothesis that the series (here residuals) under consideration contains a unit root. Numbers in parentheses are standard errors. Asterisks denote significance at 10 (*), 5 (**), and 1 (***) percent levels. Numbers highlighted in blue indicate differences from those in Table 7, either in terms of statistical significance or notable differences in magnitude.

Table A4. Parameter estimates for region-based yield indices with Prais-Winsten estimator assuming AR(1)

	EMDE	LAC	SEAO	EECA	MENA	SSA
**A. Base model**						
Constant	3527.37***	3504.51***	3374.17***	4057.70***	3138.15***	2808.97***
	(72.30)	(331.37)	(91.91)	(316.65)	(74.27)	(54.77)
Trend	102.12***	137.23***	130.84***	86.78***	65.14***	35.62***
	(2.01)	(9.08)	(2.54)	(8.81)	(2.08)	(1.53)
Adj. R^2	0.97	0.76	0.97	0.58	0.94	0.89
PP test	-4.28***	-2.78*	-3.44**	-4.28***	-7.77***	-5.34***
**B. With Trend^2**						
Constant	3678.95***	4520.06***	3431.50***	4665.23***	3180.01***	2892.32***
	(86.51)	(89.68)	(129.88)	(397.64)	(113.58)	(78.51)
Trend	87.31***	31.02***	125.04***	28.20	61.16***	27.55***
	(6.44)	(6.67)	(9.66)	(29.61)	(8.45)	(5.85)
Trend^2	0.24**	1.75***	0.09	0.94**	0.06	0.13
	(0.10)	(0.10)	(0.15)	(0.46)	(0.13)	(0.09)
Adj. R^2	0.98	0.99	0.97	0.67	0.94	0.90
PP test	-5.01***	-8.53***	-3.46**	-4.91***	-7.82***	-5.53***
**C. With a structural break in 1993**						
Constant	3636.89***	4220.34***	3380.99***	4536.86***	3189.65***	2878.61***
	(77.27)	(72.65)	(117.79)	(342.31)	(96.33)	(67.00)
Trend_before	95.07***	86.96***	130.39***	56.39***	61.93***	31.19***
	(3.56)	(3.33)	(5.45)	(15.77)	(4.42)	(3.08)
Trend_after	14.76**	110.60***	0.95	62.74**	6.76	9.31
	(6.62)	(6.20)	(10.09)	(29.32)	(8.23)	(5.73)
Adj. R^2	0.98	0.99	0.97	0.67	0.94	0.91
PP test	-4.93***	-9.04***	-3.44**	-4.95***	-7.88***	-5.58***

Notes: Yield is measured as KCal per hectare. Definitions of regional classification can be found in Table 2. Standard errors are reported in parenthesis. PP test refers to the Philips-Perron stationarity test, with the null hypothesis that the series (here residuals) under consideration contains a unit root. Numbers in parentheses are standard errors. Asterisks denote significance at 10 (*), 5 (**), and 1 (***) percent levels. Numbers highlighted in blue indicate differences from those in Table 7, either in terms of statistical significance or notable differences in magnitude.

Appendix B. Estimation and accounting for autocorrelation in the context of Box-Cox transformation

Consider the following Box-Cox regression:

$$y(\lambda) = \boldsymbol{X\beta} + u \tag{B1}$$

where y is the depedent variable, λ is the transformation parameter, $\boldsymbol{X}$ is a matrix of regressors including the constant term, $\boldsymbol{\beta}$ is the vector of parameters, and u is the vector of error term with mean 0 and variance σ^2. The Box-Cox model relies on estimating the transformation parameter λ such that:

$$y(\lambda) = \begin{cases} (y^{\lambda} - 1)/\lambda & \lambda \neq 0 \\ \log(y) & \lambda = 0 \end{cases}. \tag{B2}$$

All notations are as previously defined. Equation (B2) incorporates both a linear transformation ($\lambda = 1$) and logarithmic transformation ($\lambda = 0$) of the dependent variable. Assumingaa value of λ exists makes the error term approximately normally distributed, Box and Cox (1964) derived the likelihood function for a set of observations $\{y_1, y_2, \dots, y_T\}$) and proposed using the maximum likelihood estimation (MLE) to determine λ. The concentrated likelihood function is expressed as:

$$L(\lambda; y, X) = \frac{T}{2}(\ln(2\Pi) + 1) - \frac{T}{2}\ln\left(\hat{\sigma}^2(\lambda)\right) + (\lambda - 1)\sum_{t=1}^{T}\ln(y_t) \tag{B3}$$

where T is the number of observations, and $\hat{\sigma}^2(\lambda)$ represents the estiamted error variance for a given λ. The MLE of λ maximizes Equation (B3).

Savin and White (1978) emphasized that neglecting autocorrelation when applying the Box-Cox transformation, as in Equations (B1)-(B3), can lead to misleading results, particularly because aucorrelation may arise from misspecifciaiton of the functional form. If the error term in Equation (B1) follows an AR(1) process, we have

$$u_t = \rho u_{t-1} + \eta_t, \qquad |\rho| < 1 \tag{B4}$$

where ρ is an autoregressive coefficient, $\eta \sim iid\ N(0, \sigma^2)$.

Savin and White (1978) demonstrated that for the Box-Cox transformaiton with autocorrelated errors, as presented in Equations (B1), (B2), and (B4), the concentrated likelihood function can be written as:

$$L(\lambda, \rho; y, X) = \frac{T}{2}(\ln(2\Pi) + 1) - \frac{T}{2}\ln\left(\hat{\sigma}^2(\lambda, \rho)\right) + \frac{1}{2}\ln(1 - \rho^2) + (\lambda - 1)\sum_{t=1}^{T}\ln(y_t) \tag{B5}$$

All notations used are the same with those defined earlier. The ML estimator of λ and ρ are determined by jointly maximizing Equation (B5) over a grid of values for both parameters. Seaks and Layson (1983) propose a simplifed method for estimating the optimal transformation pramater λ in the presence of autocorrelation. They demonstrate that Equation (B5) can be expressed as:

$$L = L_1 + (\lambda - 1)\sum_{t=1}^{T}\ln(y_t) \tag{B6}$$

where $(\lambda - 1)\sum lnY_t$ represents the Jacobian of the Box-Cox transformation, and L_1 refers to the Beach-MacKinnon likelihood function, which is computed after transforming the variables by λ and applying the Beach-MacKinnon (1978) ML estimation of β and ρ. It is worth noting that the Beach-MacKinnon method is a ML procedure designed for regression models with autocorrelated errors, where β and ρ are jointly estimated.

Based on Equation (C6), Seaks and Layson (1983) propose the following process to determine the optimal λ for the Box-Cox transformation in the presence of autocorrelation:

1. Obtain $\sum logY_t$ for the dataset.
2. Iterate the following over a range of λ:
   a. Apply the Box-Cox transformation using the transformation parameter (here we only apply to the depedent variable).
   b. Conduct the Beach-MacKinnon ML estimation with the transformed Y and the independent varaibles.
   c. Retrive the log-likelihood function from step (b), denote it as L_1.
   d. Compte the Box-Cox likelihood function accounting for autocorrelation as $L = L_1 + (\lambda - 1) * \sum logY_t$, where $\sum logY_t$ is from step (1).
3. Identify the value of λ that maximizes Equation (B6).

In this analysis, our primary objective is to asses whether the aggregate yield index follows a linear or exponential growth pattern. The key transformation parameters of interest are λ=0 (logarithmic specification of yield) and λ=1 (linear specification of yield). Table B1 below presents the concentrated likelihood function for these two values of λ.

Table B1. Likelihood function Box-Cox transformation with autocorrelation: $\lambda = 0$ (log specification of yield) and $\lambda = 1$ (linear specification of yield)

	Beach-MacKinnon AR(1) Likelihood Function (L_1 in Equation (C6))				Box-Cox Likelihood Function Accounting for Autocorrelation (L in Equation (C6))			
λ	trend only	trend+ trend^2	structural break at 1993	structural break at 1978	trend	trend 2	structural break at 1993	structural break at 1978
1	-395.88	-395.82	-395.74	-395.49	-395.88	-395.82	-395.74	-395.49
0	136.40	145.07	142.41	151.29	-407.54	-398.87	-401.53	-392.65

As shown in Table B1, the results are largely consistent with those from the Box-Cox transformation without adjustments for autocorrelation, as presented in Table 4 of the main text. Except for the model assumes a structural break in 1978, the linear transformation of yield typically results in a higher likelihood than a logarithmic transformation across all other specifications.

References

Beach, Charles M., and James G. MacKinnon. "A maximum likelihood procedure for regression with autocorrelated errors." *Econometrica: Journal of the Econometric Society* (1978): 51-58.

Box, George EP, and David R. Cox. "An analysis of transformations." *Journal of the Royal Statistical Society Series B: Statistical Methodology* 26, no. 2 (1964): 211-243.

Savin, N. Eugene, and Kenneth J. White. "Estimation and testing for functional form and autocorrelation: A simultaneous approach." *Journal of Econometrics* 8, no. 1 (1978): 1-12.

Seaks, Terry G., and Stephen K. Layson. "Box-Cox estimation with standard econometric problems." *The Review of Economics and Statistics* (1983): 160-164.

Appendix C

Data on area and production are collected for 144 food commodities, including grains, oilseeds, fruits, fibers, and other crops, for 1961-2021 from FAOSTAT. The commodities considered in the analysis combined account for more than 98 percent of the global total agricultural land use during the sample period. The calorific information of most commodities is obtained from the FAO, as reported in the Food Balance Sheets.

Table A1 shows the calorific content of each crop. Specifically, the second column denotes the Item code used by FAO. The third column gives the calorific content of each commodity per kilogram, while the last two columns give the calorific content of each commodity on a hectare basis at the beginning (1961) and end (2021) of the sample.

Table C1. Calorific content of commodities (page 1)

Commodity	Code	Kcal/kg	Kcal/Ha, 1961	Kcal/Ha, 2021
Wheat	15	3,340	3,636,926	11,662,946
Rice, paddy	27	2,800	5,234,040	13,339,760
Barley	44	3,320	4,409,624	9,878,660
Maize	56	3,560	6,914,588	20,927,816
Rye	71	3,190	3,701,995	9,730,776
Oats	75	3,850	4,989,985	9,087,540
Millet	79	3,400	2,014,500	3,307,180
Sorghum	83	3,430	3,051,328	5,142,942
Buckwheat	89	3,300	1,763,520	3,111,570
Quinoa	92	3,420	2,110,824	2,623,482
Fonio	94	3,380	1,959,048	2,514,044
Triticale	97	3,270	#N/A	12,672,885
Canary seed	101	3,880	2,581,752	3,194,404
Mixed grain	103	3,400	6,649,720	11,234,960
Cereals, nes	108	3,400	2,029,120	6,064,240
Potatoes	116	670	8,184,519	13,897,542
Sweet potatoes	122	920	6,760,068	11,033,468
Cassava	125	1,090	8,070,687	11,572,203
Yautia (cocoyam)	135	1,090	5,531,532	13,459,647
Taro (cocoyam)	136	860	5,088,534	5,943,460
Yams	137	1,010	7,306,845	8,737,914
Roots and Tubers, nes	149	910	4,171,622	10,698,415
Sugar cane	156	300	15,079,500	21,169,890
Sugar beet	157	700	16,221,450	42,985,250
Sugar crops, nes	161	3,900	20,127,900	29,375,190
Beans, dry	176	3,410	1,681,812	2,631,156
Broad beans, horse beans, dry	181	3,430	3,074,309	7,513,758
Peas, dry	187	3,460	3,366,580	6,093,060
Chick peas	191	3,580	2,323,420	3,786,924
Cow peas, dry	195	3,420	1,232,568	2,060,892
Pigeon peas	197	3,430	2,801,967	2,955,288

Table C1. Calorific content of commodities (continued, page 2)

Commodity	Code	Kcal/kg	Kcal/Ha, 1961	Kcal/Ha, 2019
Lentils	201	3,460	1,826,188	3,474,878
Bambara beans	203	3,650	2,667,785	2,149,120
Vetches	205	3,250	2,800,200	6,101,875
Lupins	210	3,900	2,261,220	5,488,080
Pulses, nes	211	3,400	1,700,680	2,584,000
Brazil nuts, with shell	216	3,150	103,909,680	#N/A
Cashew nuts, with shell	217	2,520	1,376,928	1,423,548
Chestnuts	220	1,580	9,129,398	6,312,258
Almonds, with shell	221	2,360	2,430,328	4,127,876
Walnuts, with shell	222	2,890	8,903,512	8,890,507
Pistachios	223	2,890	1,691,517	3,239,112
Kolanuts	224	3,490	4,311,895	1,920,198
Hazelnuts, with shell	225	2,910	1,771,608	3,016,215
Arecanuts	226	2,450	1,746,115	3,802,890
Nuts, nes	234	2,620	16,815,422	2,977,368
Soybeans	236	3,350	3,781,145	9,613,495
Groundnuts, with shell	242	4,140	3,516,102	6,823,134
Coconuts	249	1,840	8,359,672	10,362,696
Oil palm fruit	254	1,580	5,949,964	22,757,214
Olives	260	1,750	5,504,275	3,902,500
Karite Nuts (Sheanuts)	263	5,790	11,515,152	6,865,203
Oil of castor beans*	265	4,420	2,078,726	6,344,910
Sunflower seed	267	3,080	3,149,300	6,068,524
Rapeseed	270	4,940	2,829,632	9,582,612
Tung oil*	275	2,652	21,128,219	7,228,556
Safflower seed	280	3,140	1,355,852	2,329,880
Sesame seed	289	5,730	1,639,353	2,911,413
Mustard seed	292	4,690	2,495,549	3,955,546
Poppy seed	296	5,330	2,792,387	2,922,972
Melonseed	299	4,000	2,332,400	2,070,000
Oil of kapok*	310	2,431	3,115,083	3,715,784
Seed cotton*	328	2,530	2,181,872	5,674,284
Linseed	333	4,980	1,999,968	4,014,378
Oil of hempseed*	336	4,332	1,044,012	2,110,984
Oilseeds, Nes	339	3,870	4,255,839	3,365,352
Cabbages and other brassicas	358	190	3,284,226	5,559,609
Artichokes	366	200	1,655,000	2,527,420
Asparagus	367	120	317,940	639,852
Lettuce and chicory	372	120	1,826,256	2,671,476
Spinach	373	160	1,420,864	5,609,360
Cassava leaves	378	530	#N/A	5,883,795
Tomatoes	388	170	2,793,882	6,222,255
Cauliflowers and broccoli	393	90	1,296,945	1,687,806
Pumpkins, squash and gourds	394	190	849,395	3,009,220

Table C1. Calorific content of commodities (continued, page 3)

Commodity	Code	Kcal/kg	Kcal/Ha, 1961	Kcal/Ha, 2019
Cucumbers and gherkins	397	130	1,226,004	5,597,449
Eggplants (aubergines)	399	210	1,861,944	6,277,740
Chillies and peppers, green	401	250	2,241,400	4,413,775
Onions (inc. shallots), green	402	240	2,607,864	5,071,008
Onions, dry	403	310	3,635,742	5,718,105
Garlic	406	1,300	7,249,320	22,098,310
Leeks, other alliaceous veg	407	370	4,896,839	6,103,372
Beans, green	414	500	2,641,850	7,380,150
Peas, green	417	310	1,799,364	2,456,874
String beans	423	270	1,446,957	2,594,484
Carrots and turnips	426	380	6,123,510	14,446,384
Okra	430	310	1,053,070	1,353,801
Maize, green	446	560	3,007,704	4,756,528
Mushrooms and truffles	449	240	40,281,528	#N/A
Chicory roots	459	600	10,941,300	6,034,260
Carobs	461	1,110	3,260,514	3,913,638
Vegetables fresh, nes	463	220	1,852,202	3,135,968
Bananas	486	600	6,407,520	14,050,800
Plantains	489	750	3,920,550	5,004,375
Oranges	490	340	4,306,304	6,533,270
Tangerines, mandarins, clem.	495	320	2,642,720	4,317,760
Lemons and limes	497	150	1,912,110	2,334,495
Grapefruit (inc. pomelos)	507	160	3,416,208	4,237,056
Citrus fruit, nes	512	260	1,020,916	2,438,878
Apples	515	480	4,754,832	9,271,488
Pears	521	540	5,633,712	9,900,576
Quinces	523	350	3,113,880	3,216,955
Apricots	526	450	2,778,885	2,917,845
Sour cherries	530	450	3,850,515	3,037,095
Cherries	531	650	9,040,200	3,937,505
Peaches and nectarines	534	330	3,109,722	5,481,630
Plums and sloes	536	520	8,100,560	2,400,632
Stone fruit, nes	541	520	2,937,688	4,258,124
Pome fruit nes	542	480	#N/A	3,303,600
Strawberries	544	280	2,244,284	6,593,104
Raspberries	547	470	2,898,631	3,768,507
Gooseberries	549	440	1,711,072	2,232,384
Currants	550	590	23,201,986	3,298,808
Blueberries	552	550	6,384,400	3,739,395
Cranberries	554	470	3,036,858	9,865,253
Berries Nes	558	490	3,935,141	3,133,550
Grapes	560	530	2,441,127	5,790,833
Watermelons	567	170	1,551,539	5,699,369
Other melons	568	170	1,894,905	4,515,625

Table C1. Calorific content of commodities (continued, page 4)

Commodity	Code	Kcal/kg	Kcal/Ha, 1961	Kcal/Ha, 2019
Figs	569	730	1,844,856	3,285,803
Mangoes, mangosteens, guavas	571	450	3,829,905	4,294,125
Avocados	572	1,190	10,833,165	12,044,466
Pineapples	574	260	2,697,162	7,116,044
Dates	577	1,560	11,993,280	11,570,052
Persimmons	587	820	6,633,882	3,441,622
Cashewapple	591	430	1,807,505	1,184,005
Kiwi fruit	592	520	#N/A	8,095,568
Papayas	600	260	3,012,022	7,539,506
Fruit, tropical fresh nes	603	410	2,962,332	3,149,866
Fruit Fresh Nes	619	450	3,196,440	3,299,625
Coffee, green	656	470	218,080	411,344
Cocoa beans	661	4,140	1,115,316	2,002,518
Tea	667	400	288,040	2,149,840
Mate	671	400	21,030,040	2,207,120
Pepper (Piper spp.)	687	2,760	1,284,780	3,230,580
Chillies and peppers, dry	689	3,180	2,741,160	9,499,932
Vanilla bean#	692	3,330	278,721	249,084
Cinnamon (canella)	693	2,610	1,400,787	1,940,796
Cloves	698	3,230	1,110,151	895,679
Nutmeg, mace and cardamoms	702	5,250	829,500	1,642,725
Anise, badian, fennel, corian.	711	3,450	2,301,495	4,046,505
Ginger	720	3,470	6,668,646	37,765,745
Spices, nes	723	3,370	19,963,880	7,541,386
Peppermint#	748	700	5,808,320	12,844,160

Notes: Commodity name and item code are as in the FAO database. #NA indicates either acreage or production is not available from FAO. (*) denotes calorie info has been adjusted by the following factors: Castor oil seed (0.5); Tung nuts (0.3); Kapok fruit (0.064); Seed cotton (0.61), Hempseed (0.49); (#) means it applies to Vanilla bean and Peppermint, calorie info from the U.S. Department of Agriculture.
Source: Authors, calculations based on data from the Food and Agriculture Organization and U.S. Department of Agriculture.
